# Associations of Multimodal Analgesia With Postoperative Pain Trajectories and Morphine Consumption After Hepatic Cancer Surgery

**DOI:** 10.3389/fmed.2021.777369

**Published:** 2022-01-28

**Authors:** Chia-Yi Yeh, Wen-Kuei Chang, Hsiang-Ling Wu, Gar-Yang Chau, Ying-Hsuan Tai, Kuang-Yi Chang

**Affiliations:** ^1^Department of Anesthesiology, Taipei Veterans General Hospital, Taipei, Taiwan; ^2^School of Medicine, National Yang Ming Chiao Tung University, Taipei, Taiwan; ^3^Department of Surgery, Taipei Veterans General Hospital, Taipei, Taiwan; ^4^Department of Anesthesiology, Shuang Ho Hospital, Taipei Medical University, New Taipei City, Taiwan; ^5^School of Medicine, College of Medicine, Taipei Medical University, Taipei, Taiwan

**Keywords:** hepatic cancer, postoperative pain, latent curve model, pain trajectory, multimodal analgesia

## Abstract

**Background:**

This study aimed to investigate the influential factors of postoperative pain trajectories and morphine consumption after hepatic cancer surgery with a particular interest in multimodal analgesia.

**Methods:**

Patients receiving hepatic cancer surgery at a tertiary medical center were enrolled between 2011 and 2016. Postoperative pain scores and potentially influential factors like patient characteristics and the analgesic used were collected. Latent curve analysis was conducted to investigate predictors of postoperative pain trajectories and a linear regression model was used to explore factors associated with postoperative morphine consumption.

**Results:**

450 patients were collected, the daily pain scores during the first postoperative week ranged from 2.0 to 3.0 on average. Male and higher body weight were associated with more morphine consumption (both *P* < 0.001) but reduced morphine demand was noted in the elderly (*P* < 0.001) and standing acetaminophen users (*P* = 0.003). Longer anesthesia time was associated with higher baseline pain levels (*P* < 0.001). In contrast, male gender (*P* < 0.001) and standing non-steroidal anti-inflammatory drugs (NSAIDs) use (*P* = 0.012) were associated with faster pain resolution over time.

**Conclusions:**

Multimodal analgesia with standing acetaminophen and NSAIDs had benefits of opioid-sparing and faster pain resolution, respectively, to patients receiving hepatic cancer surgery.

## Introduction

Hepatocellular carcinoma (HCC), the most frequent primary liver cancer, often derives from chronic liver disease (1). Although a variety of therapeutic options are available, surgical resection remains one of the main curative therapeutic modalities for HCC (2, 3). However, surgical liver resection, especially open hepatectomy with the use of a subcostal incision, is associated with significant postoperative pain. Adequate pain control after liver resection is essential for early mobilization and enhanced recovery (4). Inadequate postoperative pain management is related to postoperative complications and impaired clinical outcomes, prolonged length of hospital stay, increased medical costs, development of chronic pain, and worse patient quality of life (5).

Multimodal analgesia is the concept of combining different analgesic modalities that work through different mechanisms of action to achieve better pain relief while reducing opioid consumption as well as drug-related adverse effects (6–8). Since its introduction in 1993 by Kehlet and Dahl (9), multimodal analgesia has been extensively studied but few studies had ever investigated how multimodal analgesics work together to alter postoperative pain over time after hepatic cancer surgery. Accordingly, it is of interest and importance to guide clinical pain management after hepatic cancer surgery with the aid of a trajectory analytical tool like the latent curve model (10) for better interpretation of variations in postoperative pain over time (11).

In order to investigate the influential factors of postoperative pain trajectories in patients with hepatic cancer surgery, we conducted this retrospective study and employed latent curve analysis to explore factors associated with the variations in postoperative pain trajectories over time. We hypothesized that the use of multimodal analgesics, along with other patient characteristics and surgical features, would modify the postoperative pain trajectories over time and reduce opioid consumption. Furthermore, the final predictive models which best accounted for the changes in postoperative pain trajectories over time, opioid consumption, and length of hospital stay after hepatic cancer surgery would also be determined after the model selection processes.

## Materials and Methods

### Setting and Patient Selection

After the approval of the Institutional Review Board of Taipei Veterans General Hospital, (IRB-TPEVGH No. 2019-07-004BC), we conducted this retrospective study in our hospital and the inclusion criteria were patients undergoing curative surgery for stage I-III hepatocellular carcinoma with postoperative intravenous patient-controlled analgesia (IVPCA) between March 2011 and December 2016. The exclusion criteria were repeat surgery, liver transplantation for HCC, or distant metastasis diagnosed at the time of surgery. All data were extracted from our electronic medical record system by an anesthesiologist not involved in statistical analysis. Data quality was verified through random sampling by the authors.

### Postoperative Pain Management

In brief, liver cancer surgery was performed under general anesthesia with inhalation agents and neuromuscular blocking. Intravenous fentanyl 2–4 ug/kg was also given at the induction of general anesthesia and no other analgesics were used intra-operatively. IVPCA was administered after surgery *via* an infusion pump (Gemstar™ Yellow, Hospira, IL, USA) to deliver morphine with a bolus dose of 0.8–1.2 mg and a lockout interval of 6 min and typically continued for about 3 days after surgery. Acetaminophen and non-steroidal anti-inflammatory drugs (NSAIDs) might also be used for postoperative pain relief during the course of IVPCA or after the end of the IVPCA course on the 4th postoperative day.

### Pain Measurements, Data Collection, and Endpoints

The primary endpoint was postoperative pain scores evaluated using a self-report numerical rating scale (NRS) from 0 to 10, with 10 being the maximum imaginable pain and 0 indicating no pain at all, by nurses in charge at least one time per day after surgery. In this study, the mean daily NRS pain scores during the first postoperative week were collected and employed in the subsequent analyses. The secondary endpoints were morphine consumption during the first three postoperative days and length of hospital stay (LOS) after surgery. Other collected variables included comorbidities, severity of liver cirrhosis, serum aspartate aminotransferase (AST), and alanine aminotransferase (ALT). Besides, surgical features such as blood loss (log-transformed), perioperative transfusion (including packed red blood cell, fresh frozen plasma, or platelet), uses of laparoscopic or robot-assisted techniques, and anesthesia time (log-transformed) were gathered as well.

### Statistical Analysis

Patient attributes and pain scores are presented as mean with SD or count with percentage as appropriate. Right-skewed continuous variables like surgical blood loss and anesthesia time were log-transformed. Latent curve analysis with two parameters, intercept and slope, for baseline and decreasing trend of postoperative pain trajectories, respectively, was performed to evaluate how analgesic modalities and patient characteristics affected the variations in pain scores over time. A backward model selection strategy was used to identify explanatory variables of the intercept and slope parameters and determine the final multiple predictors model. The details of statistical technique on latent curve analysis refer to the previous literature (12). The root mean square error of approximation (RMSEA) was used to evaluate the model fit and the values of <0.1 indicated acceptable model fit (13). Linear regression analysis with a stepwise model selection strategy was employed to explore predictors of morphine consumption in the first three postoperative days and log-transformed LOS. All latent curve analyses were implemented using AMOS 18.0 (SPSS Inc., Chicago, IL, USA). Other statistical analyses were conducted with PASW 18.0 software (SPSS Inc., Chicago, IL, USA).

## Results

**Figure 1:**
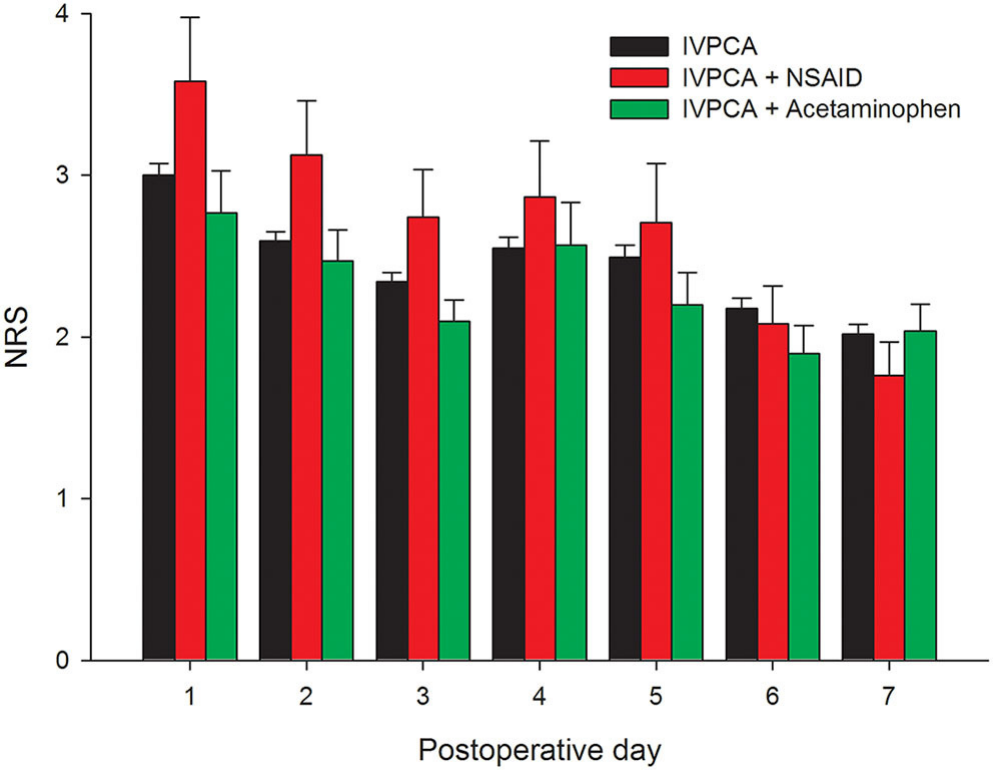
Daily mean pain scores during the first postoperative week after liver cancer surgery.

A total of 450 patients undergoing liver cancer surgery were included in the analysis and their characteristics are presented in Table 1. Note that only 7.1 and 5.6% of patients received standing acetaminophen and NSAIDs, respectively, during the IVPCA course. The mean pain scores were 3.0, 3.6, and 2.8 for patients using pure IVPCA, IVPCA combined with NSAIDs, and IVPCA combined with acetaminophen on the postoperative day (POD) 1, respectively. Afterward, the pain intensity fluctuated and decreased gradually to 2.0, 1.8, and 2.0 for these three groups on POD 7 (Figure 1). The mean morphine consumption during the first three postoperative days was 76.9 mg and the length of hospital stay after surgery was 11 days.

**Table 1:** Patient characteristics.

	**Mean, median or count**	**Standard deviation (% or IQR)**
Sex (M)	335	(74.4%)
Age (y/o)	62	12
Body weight (kg)	66.0	11.5
ASA physical status > 3	157	(34.9%)
Diabetes	119	(26.4%)
Chronic kidney disease	35	(7.8%)
Anemia	129	(28.7%)
Cirrhosis	195	(43.3%)
Laparoscopic or robotic surgery	57	(12.7%)
Surgical blood loss (ml)^*^	9.02	1.48
Anesthesia time (min)^*^	8.39	0.44
Perioperative transfusion	267	(59.3%)
Standing acetaminophen	32	(7.1%)
Standing NSAIDs	25	(5.6%)
Morphine dose (mg)	76.9	29.4
Length of hospital stay (days)	11	(9–13)

* *Log2 transformed; Perioperative transfusion includes packed red blood cell, fresh frozen plasma, or platelet*.

**Figure 2:**
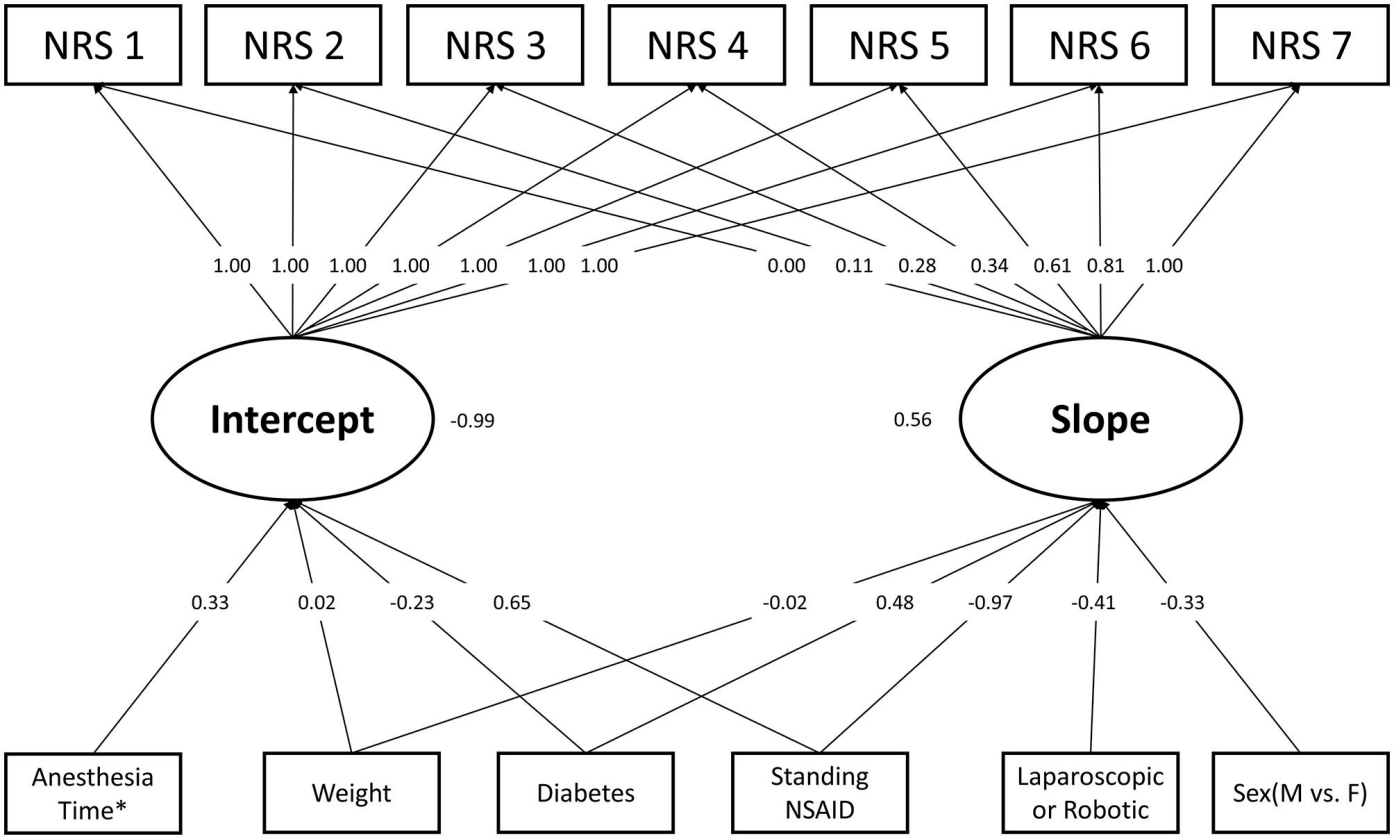
Final multiple predictors latent curve model. *Log2 transformed.

The results of the univariate analysis using the single predictor latent curve model are presented in Table 2. Notice that standing acetaminophen was not associated with intercept or slope parameters in the univariate analysis. Table 3 illustrates the results of the final model in the latent curve analysis after the backward model selection. Four independent predictors were associated with the intercept parameter of postoperative pain trajectory. Bodyweight, anesthesia time, and standing NSAIDs use exerted positive effects on intercept but diabetes is negatively associated. In contrast, five variables were significantly related to the slope parameter and diabetes exhibited the only positive association with the slope, which means a smoother decreasing trend in pain resolution. Male, higher body weight, laparoscopic or robotic surgery, and standing NSAIDs use had a negative connection to the slope parameter of postoperative pain trajectory, which implied a faster pain resolution after surgery. Based on our analytical results, the daily mean pain score during the first postoperative week can be estimated using the prediction model in Table 3. The RMSEA value of the final model was 0.08 and the graphic presentation of the final model is illustrated in Figure 2.

**Table 2:** Univariate analysis.

	**Intercept**			**Slope**		
	**Estimate**	**SE**	**p**	**Estimate**	**SE**	**p**
Sex (M vs F)	0.297	0.1	0.003	−0.571	0.146	<0.001
Age	−0.009	0.004	0.014	0.01	0.005	0.064
Body weight	0.013	0.004	<0.001	−0.017	0.005	0.002
ASA > 3	−0.006	0.086	0.949	0.01	0.128	0.937
Diabetes	−0.118	0.096	0.22	0.373	0.141	0.008
Chronic kidney disease	−0.152	0.153	0.32	0.173	0.226	0.446
Anemia	−0.025	0.091	0.787	0.187	0.134	0.165
Cirrhosis	−0.061	0.085	0.47	0.256	0.125	0.04
Laparoscopic or robotic surgery	−0.033	0.125	0.792	−0.31	0.184	0.092
Blood loss^*^	0.071	0.028	0.011	0.038	0.041	0.356
Anesthesia time^*^	0.368	0.093	<0.001	−0.035	0.139	0.803
Perioperative transfusion	0.132	0.084	0.115	0.019	0.124	0.877
Standing acetaminophen	−0.208	0.16	0.194	0.021	0.237	0.931
Standing NSAIDs	0.606	0.186	0.001	−0.891	0.274	0.001

* *Log2 transformed*.

**Table 3:** Final multiple predictors model for postoperative pain trajectory.

	**Intercept**			**Slope**		
	**Estimate**	**SE**	**p**	**Estimate**	**SE**	**p**
Sex (M vs. F)		–		−0.333	0.139	0.017
Body weight	0.015	0.004	<0.001	−0.017	0.006	0.006
Diabetes	−0.23	0.098	0.019	0.479	0.145	<0.001
Laparoscopic or robotic surgery		–		−0.407	0.168	0.016
Anesthesia time^*^	0.333	0.086	<0.001		–	
Standing NSAIDs	0.654	0.189	<0.001	−0.965	0.279	<0.001

* *Log2 transformed*.

With respect to the explanatory model of morphine consumption during the first three postoperative days, four predictors were identified after the model selection in Table 4. Note that higher age and standing acetaminophen use was negatively associated with morphine consumption after surgery but bodyweight and the male gender were connected to increasing morphine demand. Table 5 shows seven model selected predictors of length of hospital stay after liver cancer surgery and five of them associated with an increased hospital stay, including more blood loss during surgery, diabetes, older age, longer anesthesia time, and liver cirrhosis. In contrast, greater body weight had the connection to a shorter hospital stay and on average, laparoscopic or robotic surgery tended to decrease 18% of the length of hospital stay after liver cancer surgery.

**Table 4:** Predictors of morphine consumption.

	**β**	**SE**	**Standardized β**	**p**
Body weight	0.44	0.12	0.173	< 0.001
Age	−0.61	0.11	−0.247	< 0.001
Sex (M vs. F)	12.58	3.17	0.187	< 0.001
Standing acetaminophen	−15.08	5.02	−0.131	0.003
Constant	77.22	10.83		< 0.001

**Table 5:** Predictors of length of hospital stay.

	**β**	**SE**	**Standardized β**	**p**	**Exp(β)**
Surgical blood loss^*^	0.040	0.011	0.191	0.000	1.04
Laparoscopic or robotic	−0.199	0.044	−0.209	0.000	0.82
surgery					
Diabetes	0.105	0.032	0.147	0.001	1.11
Age 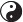	0.026	0.012	0.098	0.029	1.03
Anesthesia time^*^	0.092	0.037	0.129	0.014	1.10
Cirrhosis	0.059	0.028	0.093	0.034	1.06
Body weight 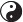	−0.025	0.012	−0.091	0.045	0.98
Constant	1.255	0.292		< 0.001	

* *Log2 transformed; 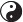
per 10 years or kilograms*.

## Discussion

In the present study, latent curve analysis was applied to model the longitudinal data of postoperative pain scores in patients receiving hepatic cancer surgery with IVPCA, and the potentially influential factors of postoperative pain trajectory were also investigated. We demonstrated that acetaminophen reduced morphine consumption during the postoperative period, whereas NSAIDs hasten pain resolution over time. When investigating the efficacy between different types of non-opioid analgesics, most studies took 24-h total morphine consumption as the primary outcome (14–17). Upon prior studies, however, some authors questioned the practicality of current conclusions of meta-analyses (18) and implied that morphine consumption may not be a good indicator for the usefulness of an analgesic (14). Since postoperative pain tends to fluctuate over time, our study evaluated pain trajectories with latent curve analysis that may better reveal the dynamic changes of postoperative pain and identified their influential factors, including patient characteristics, surgical features, and analgesic modalities in the clinical setting of hepatic cancer surgery. Furthermore, a prediction model was established to estimate pain trajectories during the first postoperative week, which provided valuable information to guide pain management after hepatic cancer surgery.

Contemporary pain management is committed to finding the best combination regimens of non-opioid analgesics as opioid-sparing strategies, yet no well-established “gold standards” for specific procedures exist currently (19). Prior meta-analyses reported the superiority of analgesic associations containing NSAIDs while comparing 24-h morphine consumption (15, 16, 20); on the other hand, the benefit of acetaminophen alone with morphine is more controversial (21–23). The combination of several nonopioid analgesics may produce an additive or even synergistic effect (14, 17). Our analysis demonstrated the independent effect of standing acetaminophen mainly exerted on the total morphine consumption but not associated with the baseline value (intercept) nor the decreasing rate (slope) of postoperative pain scores. On the contrary, standing NSAIDs showed interesting results: the independent effect not only hasten pain resolution (slope) but also increased the baseline value (intercept). A possible explanation of this finding is that patients who suffered from more severe postoperative pain tended to be administered NSAIDs as an adjunct for pain management, hence resulting in an increased baseline level of postoperative pain. In contrast, patients receiving standing acetaminophen may subjectively have neither lower baseline pain level nor faster pain resolution. Nevertheless, a decreased total morphine consumption was recognized and consequently, acetaminophen may have the potential for lowering opioid-related adverse events. In comparison, NSAIDs had no significant effect on total morphine consumption in our study.

Our study demonstrated that older patients tended to consume less morphine but aging neither affected baseline pain level nor hasten pain resolution. Prior reports showed that older age had lower pain sensitivity and better pain resolution from analgesics compared with their younger counterparts (24–26), comparing with our results. A possible explanation based on patient and surgical characteristics is that patients receiving hepatic cancer surgery were relatively younger (mean age of 62 years old) and the aging effect on postoperative pain was not a great issue in our study. Nevertheless, further investigation is necessary to unveil how aging affects pain trajectory after hepatic cancer surgery.

Some studies have addressed the relationship between body weight and postoperative pain intensity, as well as analgesic consumption, and the conclusions were conflicting (25). Our study discovered that higher body weight tended to increase morphine consumption and baseline pain level postoperatively. Furthermore, a trajectory of faster pain resolution was also found but the clinical significance of these findings should be interpreted carefully. Periasamy and colleagues reported that men undergoing intra-abdominal surgeries required more morphine in the postoperative period than women (27). Kannampallil et al. conducted a large-scale cohort study that also showed similar results (28). Our study was in line with the preceding literature. Furthermore, our analysis demonstrated that the postoperative pain score decreased faster in men, while there was no sex difference in the baseline pain level. However, a systemic review failed to determine sex as a predictor for either postoperative pain intensity or morphine consumption, which could be due to the difference in patient characteristics and sample size among separate studies (25). Note that our study included only patients receiving curative hepatic cancer surgery to reduce heterogeneity in patient features.

Several predictors of LOS after hepatic cancer surgery were identified in the current study. Among them, laparoscopic or robotic surgery showed the strongest tendency (18%) to decrease LOS after hepatic cancer surgery. The result was supported by other studies, including large-scale meta-analyses (29, 30). On the other hand, the relationship between multimodal analgesia and LOS were widely investigated in recent years. Several studies reported the advantages of multimodal analgesia toward shortening LOS in different types of surgery, including spine surgeries, colorectal surgeries, prostate surgeries, and orthopedic surgeries (31–34). However, few studies related to liver cancer surgery demonstrated this correlation (35). This may imply that LOS after liver cancer surgery is mainly determined by the patient and surgical features and the benefit of multimodal analgesia to postoperative pain relief is of limited value to further reduce LOS.

There were some limitations to the present study. First, this was an observational study and it was not possible to assess the effects of unobserved variables like preoperative pain scores, chronic analgesic medications, psychologic distress, and so on. Uncollected factors like physician preference might also be associated with the use of standing NSAIDs or acetaminophen and should be considered in future studies. Second, the study was conducted at a single medical center and the generalizability of our findings awaits further investigations. Third, the evaluation of adverse events related to multimodal analgesias, such as nausea, vomiting, sedation, gastrointestinal bleeding, and pruritus (17, 36) was beyond the scope of our study and could be considered in the future. Finally, postoperative complications which might affect outcomes (e.g., intraperitoneal hemorrhage, ascites, infection, bile leakage, thromboembolism) were not considered in the analysis due to data unavailability.

In conclusion, standing acetaminophen and NSAIDs had benefits of opioid-sparing and faster pain resolution, respectively, to patients receiving hepatic cancer surgery with IVPCA. However, their uses were not associated with LOS after hepatic cancer surgery. Latent curve analysis provided valuable information about the variations in postoperative pain trajectories over time and their influential factors. Future studies should further explore the relationships between more explanatory variables and dynamic changes in postoperative pain trajectories over time and elucidate their underlying mechanisms.

## Data Availability Statement

The original contributions presented in the study are included in the article/supplementary material, further inquiries can be directed to the corresponding author.

## Ethics Statement

The studies involving human participants were reviewed and approved by Institutional Review Board of Taipei Veterans General Hospital. Written informed consent for participation was not required for this study in accordance with the national legislation and the institutional requirements.

## Author Contributions

C-YY contributed to data acquisition and manuscript drafting. W-KC contributed to data validation and draft preparation. H-LW contributed to study coordination and data acquisition. G-YC helped review and revise the manuscript. Y-HT contributed to data acquisition. K-YC contributed to conceptualization, statistical analysis, and manuscript revision. All authors have read and agreed to the published version of the manuscript.

## Funding

This research was funded by Taipei Veterans General Hospital (V109C-063), Anesthesiology Research and Development Foundation, Taipei, Taiwan (ARDF10902), Yen Tjing Ling Medical Foundation, Taipei, Taiwan (CI-109-29), and Ministry of Science and Technology, Taipei, Taiwan, R.O.C. (MOST109-2511-H-075-003-MY2).

## Conflict of Interest

The authors declare that the research was conducted in the absence of any commercial or financial relationships that could be construed as a potential conflict of interest.

## Publisher's Note

All claims expressed in this article are solely those of the authors and do not necessarily represent those of their affiliated organizations, or those of the publisher, the editors and the reviewers. Any product that may be evaluated in this article, or claim that may be made by its manufacturer, is not guaranteed or endorsed by the publisher.

## References

[1] Forner A Reig M Bruix J. Hepatocellular carcinoma. Lancet. (2018) 391:1301–14. 10.1016/S0140-6736(18)30010-2 29307467

[2] Lurje I Czigany Z Bednarsch J Roderburg C Isfort P Neumann UP. Treatment Strategies for Hepatocellular Carcinoma (-) a Multidisciplinary Approach. Int J Mol Sci. (2019) 20. 10.3390/ijms20061465 30909504 PMC6470895

[3] Yin Z Fan X Ye H Yin D Wang J. Short- and long-term outcomes after laparoscopic and open hepatectomy for hepatocellular carcinoma: a global systematic review and meta-analysis. Ann Surg Oncol. (2013) 20:1203–15. 10.1245/s10434-012-2705-8 23099728

[4] Dieu A Huynen P Lavand'homme P Beloeil H Freys SM Pogatzki-Zahn EM. *Pain management after open liver* resection: Procedure-Specific Postoperative Pain Management (PROSPECT) recommendations. Reg Anesth Pain Med. (2021) 46:433–45. 10.1136/rapm-2020-101933 33436442 PMC8070600

[5] Bonnet F Marret E. Postoperative pain management and outcome after surgery. Best Pract Res Clin Anaesthesiol. (2007) 21:99–107. 10.1016/j.bpa.2006.12.007 17489222

[6] Buvanendran A Kroin JS. Multimodal analgesia for controlling acute postoperative pain. Curr Opin Anaesthesiol. (2009) 22:588–93. 10.1097/ACO.0b013e328330373a 19606021

[7] Corletto F. Multimodal and balanced analgesia. Vet Res Commun. (2007) 31:59–63. 10.1007/s11259-007-0085-5 17682848

[8] White PF. Multimodal analgesia: its role in preventing postoperative pain. Curr Opin Investig Drugs. (2008) 9:76–82. 18183534

[9] Kehlet H Dahl JB. The value of “multimodal” or “balanced analgesia” in postoperative pain treatment. Anesth Analg. (1993) 77:1048–56. 10.1213/00000539-199311000-00030 8105724

[10] Tai YH Wu HL Lin SP Tsou MY Chang KY. Influential factors of postoperative pain trajectories in patients receiving intravenous patient-controlled analgesia: a single-centre cohort study in Taiwan. BMJ Open. (2019) 9:e031936. 10.1136/bmjopen-2019-031936 31699739 PMC6858203

[11] Chapman CR Donaldson GW Davis JJ Bradshaw DH. Improving individual measurement of postoperative pain: the pain trajectory. J Pain. (2011) 12:257–62. 10.1016/j.jpain.2010.08.005 21237721 PMC3052945

[12] Lo PH Tsou MY Chang KY. Modeling the trajectory of analgesic demand over time after total knee arthroplasty using the latent curve analysis. Clin J Pain. (2015) 31:776–81. 10.1097/AJP.0000000000000172 25370137

[13] Bollen KA Curran PJ. *Latent curve* models: A structural equation perspective. John Wiley and Sons. (2006). 10.1002/047174609625855820

[14] Elia N Lysakowski C Tramèr MR. Does multimodal analgesia with acetaminophen, nonsteroidal antiinflammatory drugs, or selective cyclooxygenase-2 inhibitors and patient-controlled analgesia morphine offer advantages over morphine alone? Meta-analyses of randomized trials. Anesthesiology. (2005) 103:1296–304. 10.1097/00000542-200512000-00025 16306743

[15] McDaid C Maund E Rice S Wright K Jenkins B Woolacott N. Paracetamol and selective and non-selective non-steroidal anti-inflammatory drugs (NSAIDs) for the reduction of morphine-related side effects after major surgery: a systematic review. Health Technol Assess. (2010) 14:1–153. 10.3310/hta14170 20346263

[16] Martinez V Beloeil H Marret E Fletcher D Ravaud P Trinquart L. Non-opioid analgesics in adults after major surgery: systematic review with network meta-analysis of randomized trials. Br J Anaesth. (2017) 118:22–31. 10.1093/bja/aew391 28039239

[17] Beloeil H Albaladejo P Sion A Durand M Martinez V Lasocki S. Multicentre, prospective, double-blind, randomised controlled clinical trial comparing different non-opioid analgesic combinations with morphine for postoperative analgesia: the OCTOPUS study. Br J Anaesth. (2019) 122:e98–e106. 10.1016/j.bja.2018.10.058 30915987

[18] Joshi GP Kehlet H Group PW. Guidelines for perioperative pain management: need for re-evaluation. Br J Anaesth. (2017) 119:703–6. 10.1093/bja/aex304 29121311

[19] Dahl JB Nielsen RV Wetterslev J Nikolajsen L Hamunen K Kontinen VK. Post-operative analgesic effects of paracetamol, NSAIDs, glucocorticoids, gabapentinoids and their combinations: a topical review. Acta Anaesthesiol Scand. (2014) 58:1165–81. 10.1111/aas.12382 25124340

[20] Du X Gu J. The efficacy and safety of parecoxib for reducing pain and opioid consumption following total knee arthroplasty: A meta-analysis of randomized controlled trials. Int J Surg. (2018) 59:67–74. 10.1016/j.ijsu.2018.09.017 30292001

[21] Aryaie AH Lalezari S Sergent WK Puckett Y Juergens C Ratermann C. Decreased opioid consumption and enhance recovery with the addition of IV Acetaminophen in colorectal patients: a prospective, multi-institutional, randomized, double-blinded, placebo-controlled study (DOCIVA study). Surg Endosc. (2018) 32:3432–8. 10.1007/s00464-018-6062-y 29352454

[22] Hilleman DE Malesker MA Aurit SJ Morrow L. Evidence for the Efficacy of an Opioid-Sparing Effect of Intravenous Acetaminophen in the Surgery Patient: A Systematic Review. Pain Med. (2020) 21:3301–13. 10.1093/pm/pnaa256 32869091

[23] Rømsing J Møiniche S Dahl JB. Rectal and parenteral paracetamol, and paracetamol in combination with NSAIDs, for postoperative analgesia. Br J Anaesth. (2002) 88:215–26. 10.1093/bja/88.2.215 11878655

[24] Tighe PJ Le-Wendling LT Patel A Zou B Fillingim RB. Clinically derived early postoperative pain trajectories differ by age, sex, and type of surgery. Pain. (2015) 156:609–17. 10.1097/01.j.pain.0000460352.07836.0d 25790453 PMC4367128

[25] Ip HY Abrishami A Peng PW Wong J Chung F. Predictors of postoperative pain and analgesic consumption: a qualitative systematic review. Anesthesiology. (2009) 111:657–77. 10.1097/ALN.0b013e3181aae87a 19672167

[26] Gerbershagen HJ Pogatzki-Zahn E Aduckathil S Peelen LM Kappen TH van Wijck AJ. Procedure-specific risk factor analysis for the development of severe postoperative pain. Anesthesiology. (2014) 120:1237–45. 10.1097/ALN.0000000000000108 24356102

[27] Periasamy S Poovathai R Pondiyadanar S. Influences of gender on postoperative morphine consumption. J Clin Diagnostic Res. (2014) GC04. 10.7860/JCDR/2014/10770.5319 25653963 PMC4316269

[28] Kannampallil T Galanter WL Falck S Gaunt MJ Gibbons RD McNutt R. Characterizing the pain score trajectories of hospitalized adult medical and surgical patients: a retrospective cohort study. Pain. (2016) 157:2739–46. 10.1097/j.pain.0000000000000693 27548045 PMC5113285

[29] El-Gendi A El-Shafei M El-Gendi S Shawky A. Laparoscopic Versus Open Hepatic Resection for Solitary Hepatocellular Carcinoma Less Than 5 cm in Cirrhotic Patients: A Randomized Controlled Study. J Laparoendosc Adv Surg Tech. (2018) 28 A:302–310. 10.1089/lap.2017.0518 29172949

[30] Jiang B Yan XF Zhang JH. Meta-analysis of laparoscopic versus open liver resection for hepatocellular carcinoma. Hepatol Res. (2018) 48:635–63. 10.1111/hepr.13061 29330919

[31] Duellman TJ Gaffigan C Milbrandt JC Allan DG. Multi-modal, pre-emptive analgesia decreases the length of hospital stay following total joint arthroplasty. Orthopedics. (2009) 32:167. 10.3928/01477447-20090301-08 19309064

[32] Ben-David B Swanson J Nelson JB Chelly JE. Multimodal analgesia for radical prostatectomy provides better analgesia and shortens hospital stay. J Clin Anesth. (2007) 19:264–8. 10.1016/j.jclinane.2006.12.003 17572320

[33] Bohl DD Louie PK Shah N Mayo BC Ahn J Kim TD. Multimodal Versus Patient-Controlled Analgesia After an Anterior Cervical Decompression and Fusion. Spine. (2016) 41. 10.1097/BRS.0000000000001380 26679869

[34] De Roo AC Vu JV Regenbogen SE. Statewide Utilization of Multimodal Analgesia and Length of Stay After Colectomy. Journal of Surgical Research. (2020) 247:264–70. 10.1016/j.jss.2019.10.014 31706540 PMC7028497

[35] Zhou H Jia WD Qiao XF Liu FP Chen L Hu CL. [Clinical values of multimodal preventive analgesia in patients with partial hepatectomy for liver cancer]. Zhonghua Wai Ke Za Zhi. (2017) 55:141–5. 28162215 10.3760/cma.j.issn.0529-5815.2017.02.013

[36] Maund E McDaid C Rice S Wright K Jenkins B Woolacott N. Paracetamol and selective and non-selective non-steroidal anti-inflammatory drugs for the reduction in morphine-related side-effects after major surgery: a systematic review. Br J Anaesth. (2011) 106:292–7. 10.1093/bja/aeq406 21285082

